# An ultra-low-cost and adjustable in-house electrospinning machine to produce PVA nanofiber

**DOI:** 10.1016/j.ohx.2022.e00315

**Published:** 2022-05-08

**Authors:** Ika Dewi Wijayanti, Ari Kurniawan Saputra, Faris Ibrahim, Amaliya Rasyida, Putu Suwarta, Indra Sidharta

**Affiliations:** a Department of Mechanical Engineering, Institut Teknologi Sepuluh Nopember (ITS), Surabaya 60111, Indonesia‘; b Department of Material and Metallurgy, Institut Teknologi Sepuluh Nopember (ITS), Surabaya 60111, Indonesia

**Keywords:** Polymer nanofiber, Affordable electrospinning machine, Home-made electrospinning machine

## Abstract

Electrospinning is a method that uses a high-voltage electric field to fabricate nanofiber by charging and ejecting a polymer solution through a syringe. Compared to other methods, it produces nanofiber using simple and easy techniques. The widespread usage of the commercial electrospinning machines (Spinboxsystems)-BASIC KIT type for beginners is limited due to its price of over USD15,595. Additionally, the Original Equipment Manufacturer (OEM) spare parts are expensive to replace, which increases the production cost of Polyvinyl Alcohol (PVA) polymer nanofiber and hinders its application in various fields. This led to the successful design and development of an in-house built electrospinning machine at a total cost below USD2,000. The new machine is easy and simple to operate while also producing PVA nanofiber with excellent properties.

## Graphical abstract

**Figure:**
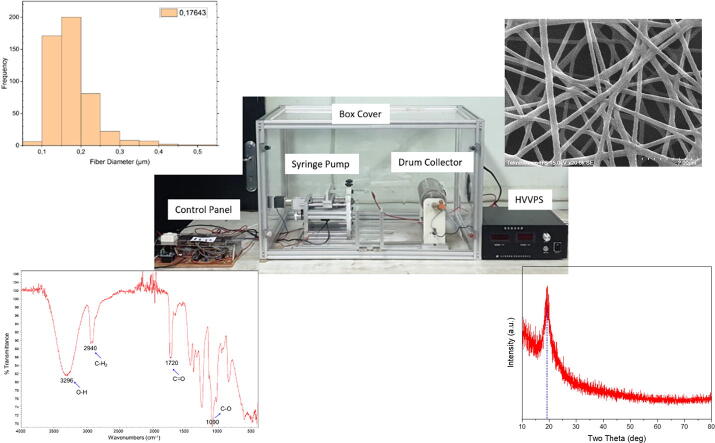

**Specifications table:** 

Hardware name	*Ultra-low-cost electrospinning machine*
Subject area	• Engineering and materials science Chemistry and biochemistry Medical, such as pharmaceutical science Biological sciences, including microbiology and biochemistry
Hardware type	• Biological sample handling and preparation Mechanical engineering and materials science
Open Source License	CC BY 4.0
Cost of Hardware	USD1,1611
Source File Repository	https://doi.org/10.17632/vzmd24hzhm.1

## Hardware in context

Electrospinning is the most effective and widely employed processing technique for the fabrication of nanofibers. It involves using an electrically charged precursor consisting of a syringe to accommodate the solution, 2 electrodes, and a DC supply voltage within the kV range [1], [2], [3], [4]. This method utilizes a potential gradient arising from the applied charge on the liquid to the collector. A high voltage is applied to dispense the solution from the syringe, thereby allowing the evaporated polymer to solidify, forming nanofiber [5]. The bond chains prevent the cooling and hardening of the resulting product, which is collected on a grounded target surface.

According to Tucker et al., several studies have been globally conducted on developing electrospun nanofiber more than four centuries ago [6]. Electrospun nanofiber possesses attractive properties, including high aspect ratio, large surface area and excellent mechanical performance, which has attracted great attention since the end of the 20th century [7], [8]. This has also led to the widespread application of nanofiber in electronics equipment, functional materials and energy storage, polymer, tissue and engineering medicine, etc. In addition, Ramakrishna reported that more than 200 universities and research institutes globally have been working on the electrospinning process [4], whereas approximately 45 published articles, including patents and papers. This number has increased dramatically to over 600 in less than a decade [9]. However, this is not surprising as this technique offers a combination of unlimited materials to fabricate nanofibers at a relatively high production rate, with great benefit to the self-controllable properties and parameters.

The interest in studying and evaluating nanofiber production using the electrospinning process has significantly grown due to the remarkable features offered by the machine. Electrospinning to produce nanofiber is a promising research topic in Indonesia, especially its application in energy storage electrodes, considering the limited and slow development of Ni-MH battery electrodes. Commercial electrospinning machines cost approximately USD15,595, which is quite expensive for local universities. An in-house built apparatus is quite affordable and is needed to boost the production rate. However, it needs to be adjustable for different input parameters to be able to produce high-quality electrospun nanofiber.

The superior properties are dependent on the successful collaboration between the processing parameters and the precursor solutions used during fabrication. The process to determine the optimal combination to produce electrospun nanofibers with enhanced properties is quite complex and outside the scope of this research. Meanwhile, to ascertain the repeatability and reproducibility of these attributes, the fabricated product from the in-house built electrospinning machine is characterized by employing different techniques such as Scanning Electron Microscopy (SEM), X-Ray Diffraction (XRD), Fourier-transform infrared spectroscopy (FTIR), and tensile test. An affordable machine produces high-quality electrospun nanofiber and boosts the country's development of material science and technology.

To summarize, the novelty of this method compared to other methods is explained as follow:

1) The machine is easily adjustable by having the self-made controller.
2) Indeed, the rotating capacity of 12,000 RPM causes this machine to be flexible and reliable for nanofiber fabrication, especially the ability to control fiber alignment.
3) For the syringe holder, this machine is customized into it 3 slots, and a crossbar combined with a lead screw serves as a locking mechanism. The holder is compatible with 3–20 ml syringes, supporting flexibility during nanofiber fabrication.
4) 30 × 30 aluminum profile used as a railing system, thereby making it easier to adjust the distance between the syringe and the collector.
5) A lid with a hinged handle made of acrylic material, used for opening and closing and to minimize the risk of being electrocuted.
6) The cover is lightweight, moveable and easily cleaned.
7) A smooth operation can be reached by using Arduino support.
8) The controllable morphology of nanofiber can be achieved by using electrospinning fabrication method.

## Hardware description

Irrespective of the fact that this electrospinning machine utilizes ultra-cheap components, it is effectively used to fabricate nanofibers. There are 2 DC motors, one serves as the syringe's plunger while the other is for winding the nanofiber collector installed on the machine. Additionally, a Nema 17HS4401-PG518 stepper motor was used as the injection propulsion component due to its high torque, excellent speed precision, and high power. The RS775-12V type DC motor was installed at the collector with a portable size, which does not affect the design of the machine. Indeed, the rotating capacity of 12,000 RPM causes this machine to be flexible and reliable for nanofiber fabrication, especially the ability to control fiber alignment.

For the syringe holder, this machine is customized into it 3 slots, and a crossbar combined with a lead screw serves as a locking mechanism. The holder is compatible with 3–20 ml syringes, supporting flexibility during nanofiber fabrication. The starting process involves incorporating 3 different precursor solutions and varying syringe sizes. It depicts the strength of this machine, especially relating to the research carried out on nanofibers with a 30 × 30 aluminum profile used as a railing system, thereby making it easier to adjust the distance between the syringe and the collector.

This machine is also equipped with a lid with a hinged handle made of acrylic material, used for opening and closing and to minimize the risk of being electrocuted, thereby making it as safe as possible for humans. Moreover, this cover is lightweight, moveable and easily cleaned. The machine has operating modes supported by Arduino, which allows for smooth operation. It is simple to set up the electrospinning process parameters by inputting 1 or 2 fingers. The procedure includes setting the needle size, flow speed, and overriding mechanism to adjust the position of the syringe plunger as required, and finally, the start and stop buttons are used to control the process.

In the case of high voltage, shoes or sandals should be used to minimize static electricity. Before mixing the precursor, the safety data sheet of the solvents should be understood. Since our electrospinning is equipped with a cover made of acrylic, when the machine is mainly in operating mode, the spread of solvent droplets from the syringe can be minimized. When finished, the inside of the acrylic cover can be cleaned with alcohol. In addition, masks and googles are highly recommended to wear. Lab coats can be added for wear.

A suitable electrospinning machine designed for nanofiber fabrication is purchased at USD 2,000, a great achievement compared to the commercial-grade ones that are expensive and cost relatively USD 15,595 with the following specifications based on the components and electrical systems used:

• Input Voltage 220 V AC
• Maximum Rotation Speed of Drum Collector 1,500 rpm
• Maximum High Voltage Power Supply 30 kV
• Syringe Size Range 3 ml, 5 ml, 10 ml, 20 ml
• Flow rate regulation 0.1 ml/h ∼ 20 ml/h
• The maximum area of nanofiber products is 200 mm × 314 mm
• Number of Syringes (parallel) 1 ∼ 3 syringes

Syringe needle to Drum collector distance 100 mm ∼ 250 mm.

The following are some of the important points from the machine:

• Nanofiber fabrication converts polymer solutions to strands.
• General Knowledge is used as a learning tool due to its simple variable process parameter settings.
• Multi-Solution Processing processes three different polymer solutions with the same parameters.

When it comes to nanotechnology, an improvement of the properties can be achieved by designing nanoscale materials, especially nanofiber. The excellent mechanical and physical properties of nanofiber including large surface to volume ratio, high porosity, controllable morphology, high chemical, and thermal stability led researchers to consider the benefits of working with nanofibers [10]. Indeed, the number of polymers responsible for the fabrication of nanofiber is large considering the compatibility between nanofiber properties and applications. One of them is Polyvinyl alcohol (PVA), a biocompatible and highly hydrophilic semicrystalline polymer with excellent properties such as strength, solubility in water, gas permeability, and thermal characteristics.

In general, nanoscale materials can be designed to exhibit improvements in their properties. Nanofiber presents several attractive properties such as superior mechanical properties, large surface area to volume ratio, and high porosity [1]. In addition, Polyvinyl alcohol (PVA) is a biocompatible and highly hydrophilic semicrystalline polymer with excellent properties such as strength, water solubility, gas permeability, and thermal stability characteristics [11]. Nanofiber fabrication using PVA based precursors through electrospinning technique has been extensively studied since in the 15th century for the preparation of ultrafine separation filters, biodegradable mats, and inorganic fibres [12].

Various types of nanofiber polymers made by electrospinning method can be categorized into three types, namely natural, synthetic, and composite polymers according to [13]. Each type of polymer determines the characteristics of the electrospun nanofiber for use in a particular application. Natural polymers such as proteins, nucleic acids, and lipids have excellent biocompatibility properties when attached to human cells. When working with synthetic polymers, such as Polyurethane (PU) and Polyvinyl alcohol (PVA), superiority in mechanical properties and degradability are very important to consider. Indeed, by combining the two polymers, the collaborative properties of biocompatibility and mechanical properties can be adjusted in several engineering applications.

In terms of energy storage applications, the achievement of improved performance can be reached by developing new energy materials derived from new nanotechnologies. In addition to the electrospinning process, various synthesis processes have been carried out on composite polymers with advanced structures and excellent properties. According to [14], hydrothermal preparation was applied on Ag doping ZnO nanoparticles for semiconductor applications. Another research by [15] revealed that Au/ZnO/RGO nanohybrids using 1,8-diamino-3,6-dioxaoctan can be successfully prepared by hydrothermal as novel functional agent photo-degradation water treatment. In case of adsorbing heavy metals, a low-cost nano-powder adsorbent from the nature, beans peel, was succeeded to remove heavy metal Cd (II) from aqueous solution [16]. Another study showed that a combination of natural extraction and hydrothermal processes which was applied to the S- and N-Codoped Carbon nanospheres can enhance the adsorption properties of Pb (II) from aqueous solutions [17]. When it comes to catalytic applications, immobilization method was used to deposit Platinum nanoparticles on reduced graphene oxide to modify the catalytic properties [18]. While according to [19], nanocomposite Pd/rGO catalyst as an efficient catalyst was synthesized sol immobilization method of deposition Pd nanoparticles on rGO. Apart from those studies, according to [19], Pd/rGO nanocomposite as an efficient catalyst was synthesized by the sol immobilization method of Pd nanoparticle deposition on rGO. Indeed, a new double perovskite Tb_2_ZnMnO_6_ nanoparticles were successfully synthesized by a sol–gel auto combustion method to increase the photocatalytic activity [20]. Another alternative in preparing the nanostructure was successfully reported by [21], [22] respectively. An eco-friendly green synthesis was used to prepare ZNO/GQD nanocomposites by extracting grafts onto Protoparmeliopsis muralis to inhibit bacterial growth in medical devices. A sonochemical route synthesis was used to prepare a novel CuI-PbI_2_ nanocomposite which produce excellent hydrogen capacity.

In the case of nanofiber fabrication, precursor was prepared using a fully hydrolysed Poly Vinyl Alcohol (PVA), which was purchased from MERCK with approx. 60,000 MW. An 8 wt% PVA was stirred with 50 ml distilled water at 80 °C until homogeneously dissolved at 200 rpm. The solution was then cooled at room temperature for 30 min. To ensure homogeneity of mixing between polymer and distilled water, the solution was afterwards stirred at 150 rpm for 15 min. The precursor was collected with a syringe which was placed on its clamp. A sheet of aluminium foil was wrapped on a collector as a nanofiber extraction site. A 5 ml disposable syringe with G-23 × 1″ needle was used. Process parameters which include voltage, flow rate, drum rotational speed, and tip-to-collector distance of 15 kV, 2 ml/hour, 200 rpm, and 15 cm respectively, were applied to the precursor.

**Fig. 1:**
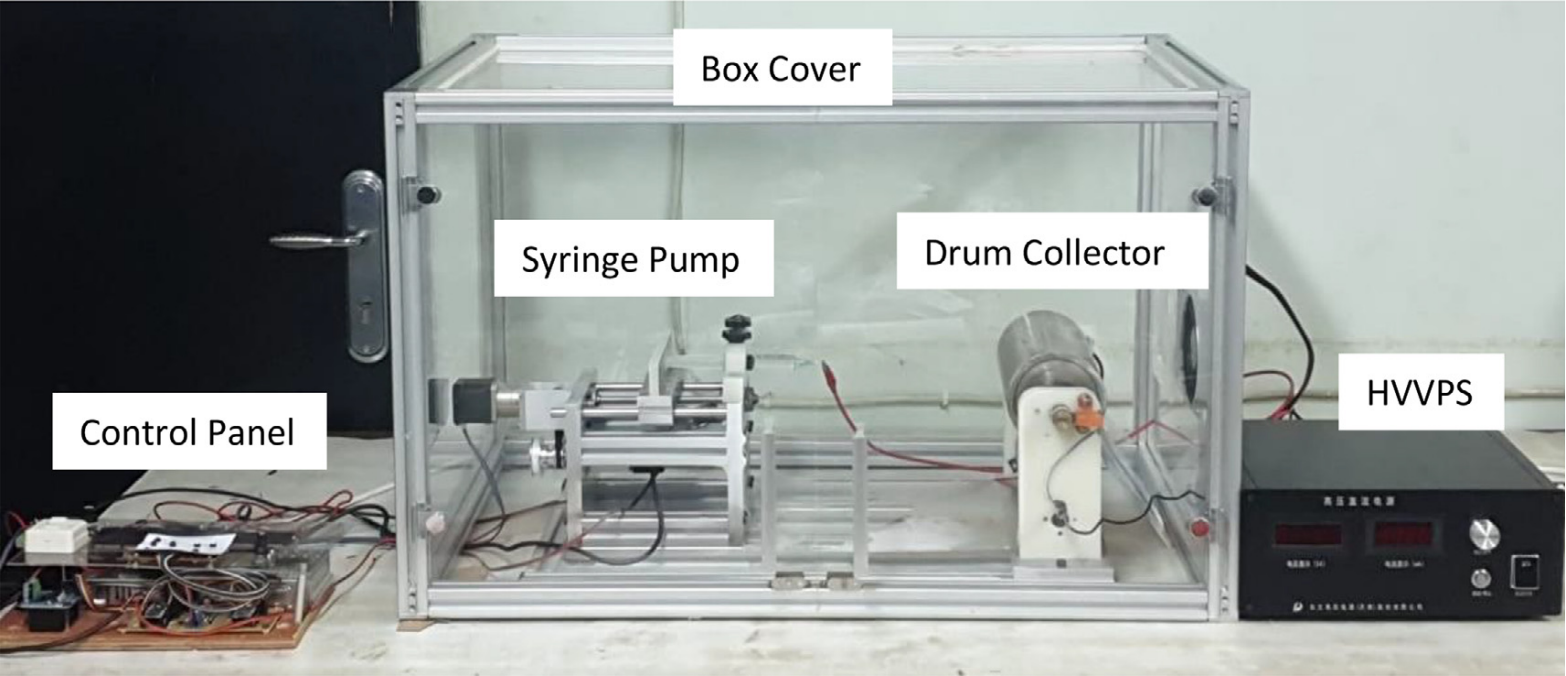
Electrospinning machine.

The ultra-cheap electrospinning machine consists of 5 main parts, namely High Voltage Variable Power Supply (HVVPS), Syringe Pump, Drum Collector, Control panel and Box Cover which can be seen in Fig. 1. This tool is built with an aluminum profile 30 × 30, the mainframe combined with aluminum plates, nylon, and acrylic. Its use aims to simplify the manufacturing (cutting and drilling) and tool disassembly processes to modify the machine according to the added advanced features.

### High Voltage Variable Power Supply (HVVPS)

The High Voltage Variable Power Supply (HVVPS) used in this system is a low-cost power supply made in China Dongwen with High Voltage Power DW-P303-1ACD1 series. This power supply includes tax and postage cost USD 838, much cheaper than other brands that can reach a price of USD2,500 and are capable of producing DC voltage output from 0 V to 30 kV with a maximum current of 1 mA. The detailed specifications of the high-voltage variable power supply are shown in Table 1.

**Table 1:** High voltage power supply specification.

‘Price	USD 700
Model number	DW-P303-1ACD1
Input voltage	AC 220 V ± 10%
Output voltage	DC 0∼+30,000 V
Output current	1 mA
Dimensions	300*270*120 mm (Aluminum case, the outer lead length is 1.5 m)
Grounding method:	AC isolation, the reference ground is connected to the earth
Protection mode	Current limiting type
Adjustment method	Potentiometer adjustment
Time drift accuracy	0.1%/hour (Calculated after 30 min)
Temperature drift accuracy	0.1%/°C
Load regulation rate	0.5%
Working temperature	−10 °C∼+50 °C

### Syringe pump

**Fig. 2:**
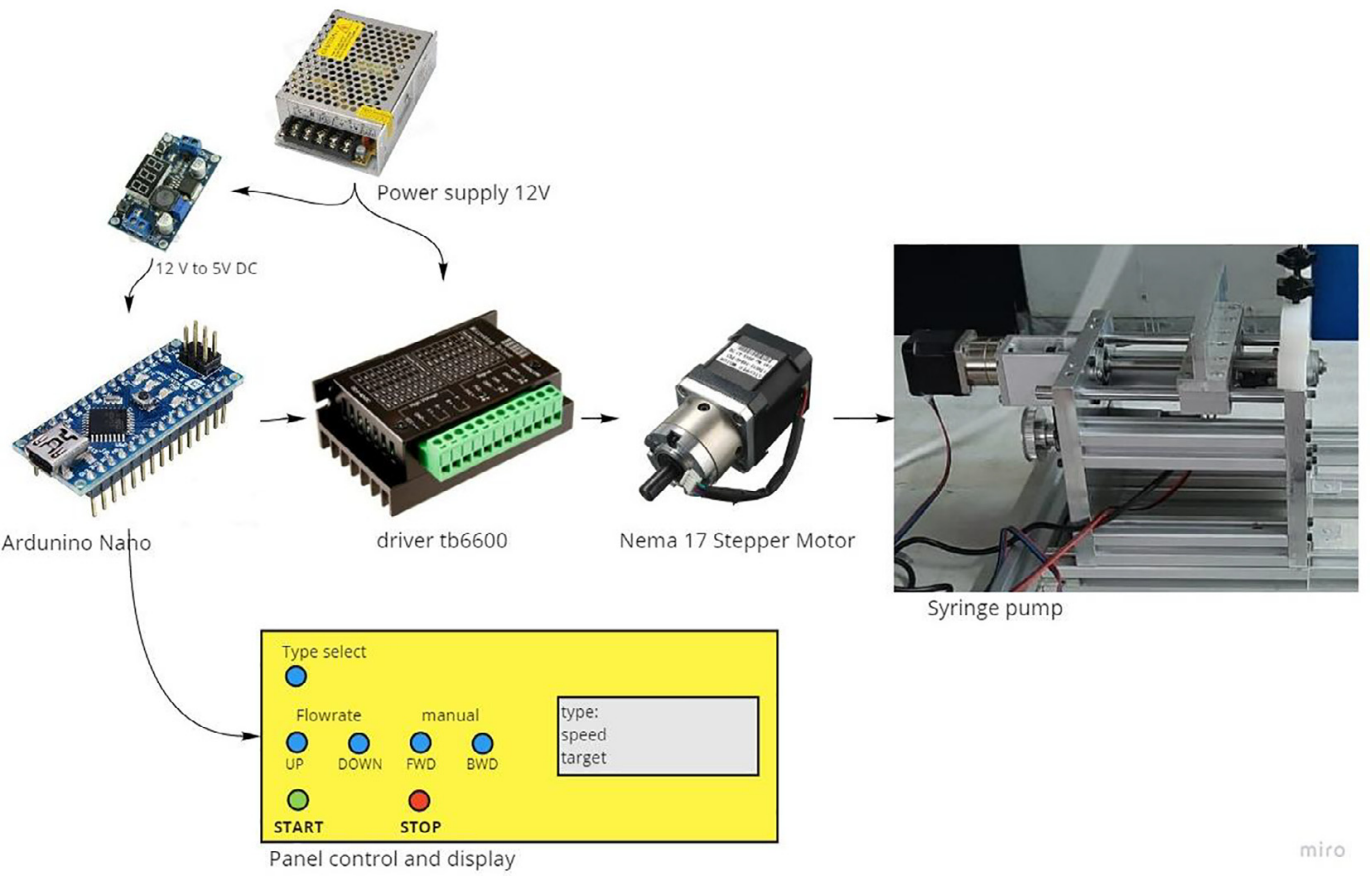
Hardware and electronic system of Syringe Pump.

As seen in Fig. 2, the syringe pump comprises 2 aluminum plates and a 30 × 30 profile frame. Due to the easy manufacturing process, these materials were selected, including stable movement during the injection. This pump is installed with 3 syringes placed in a parallel position, and it costs USD 265, which is relatively 65% cheaper than the market price (∼USD 800). For the driving mechanism, a Nema 17 stepper motor with Gearbox PG518 and a reduction ratio of 1:5.18 was connected to the lead screw with a diameter and pitch of 8 mm and 1 mm, respectively, to drive the syringe. The TB6600 micro-stepping driver was set at a resolution of 1/32 (6,400 pulses/rev) to ascertain the accuracy of the feeding pump, which was realized to be quite high, 6,400 pulses × 5.18 = 33,163 pulses/mm. A Maxwell 12 V 4.2A power supply was used as the DC voltage source while, an Arduino Nano with a control panel and display to set the menu and feeding scheme served as the controller. Fig. 2 shows a schematic of the syringe pump hardware system.

### Drum collector

**Fig. 3:**
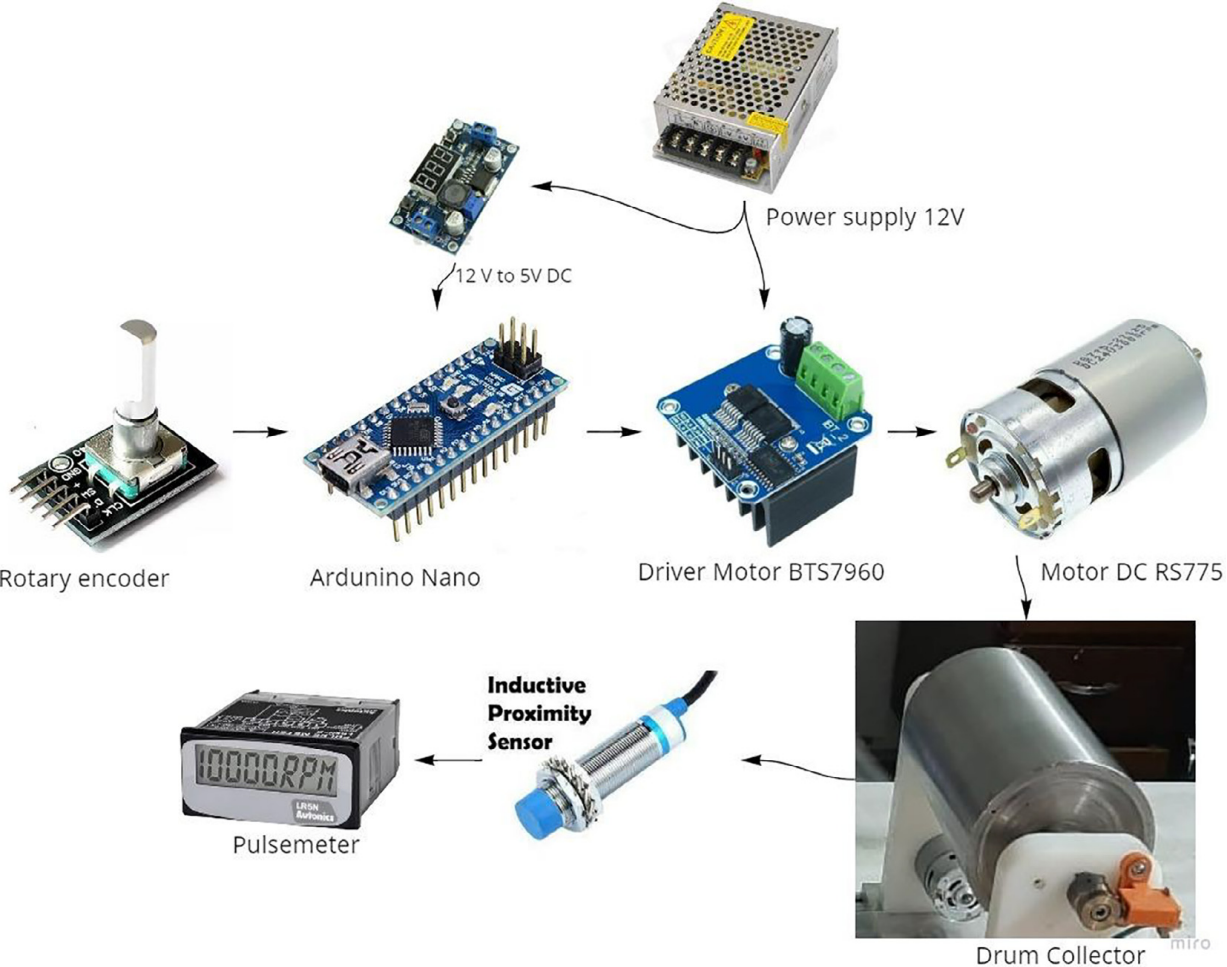
Hardware and electronic system of drum collector.

The drum collector which can be seen in Fig. 3 has a diameter and length of 100 mm and 200 mm, respectively, thereby obtaining product specimens measuring 200 × 314 mm. This material is made of SS304 due to its excellent corrosive resistance. The RS775 DC motor is used to drive the drums connected by a belt and pulley in the ratio of 1:2 and enables it to be adjusted from 0 to 1,500 pm. The rotation of the motor is controlled by an Arduino Nano connected to a rotary encoder and an Autonics Pulsemeter, which serves as a speed control input and monitor for displaying the rotational speed of the drum collector. Fig. 3 shows a schematic of the Drum Collector hardware and system.

## Design files

### Mechanical components

**Fig. 4:**
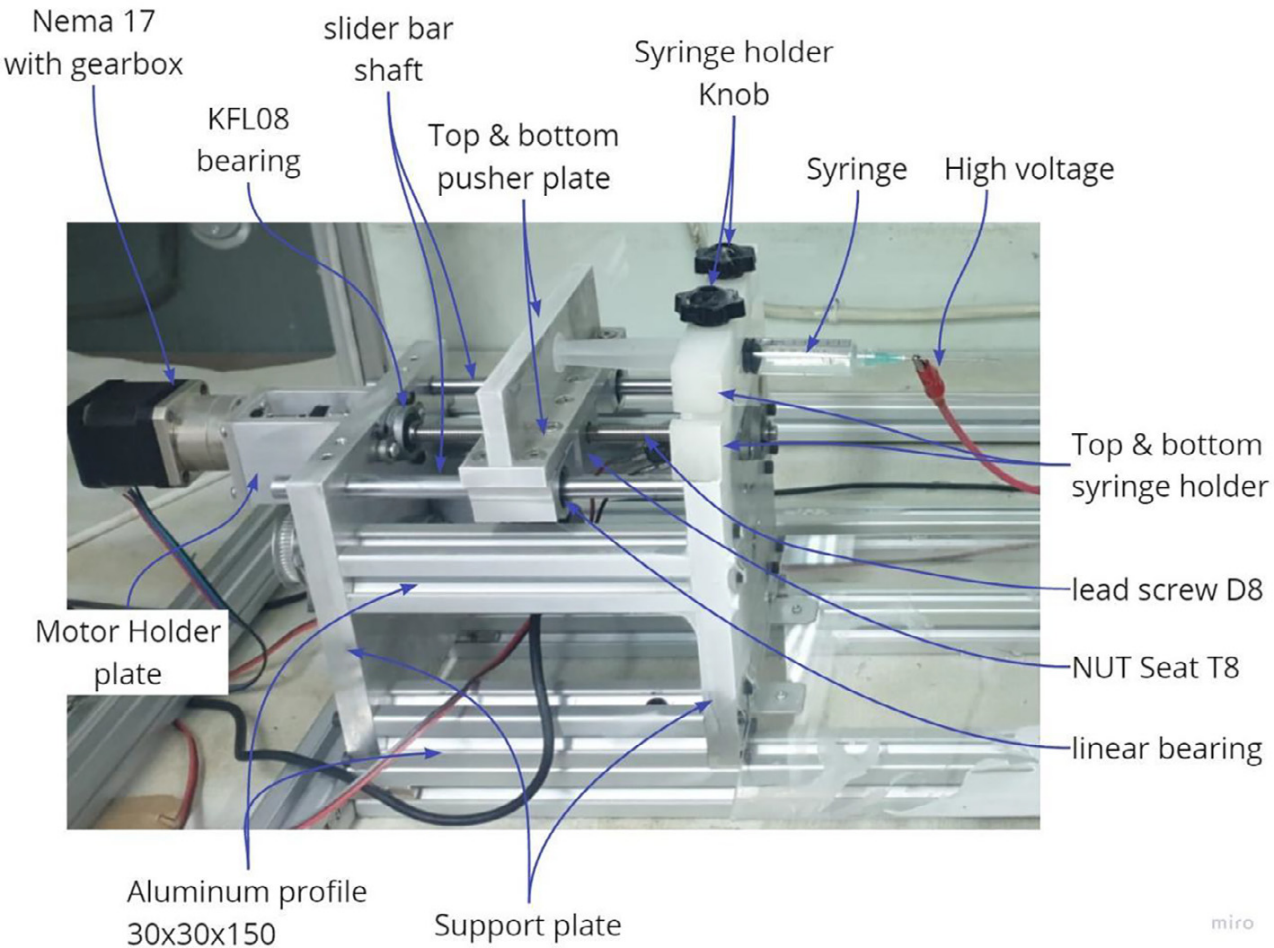
Syringe pump components.

**Fig. 5:**
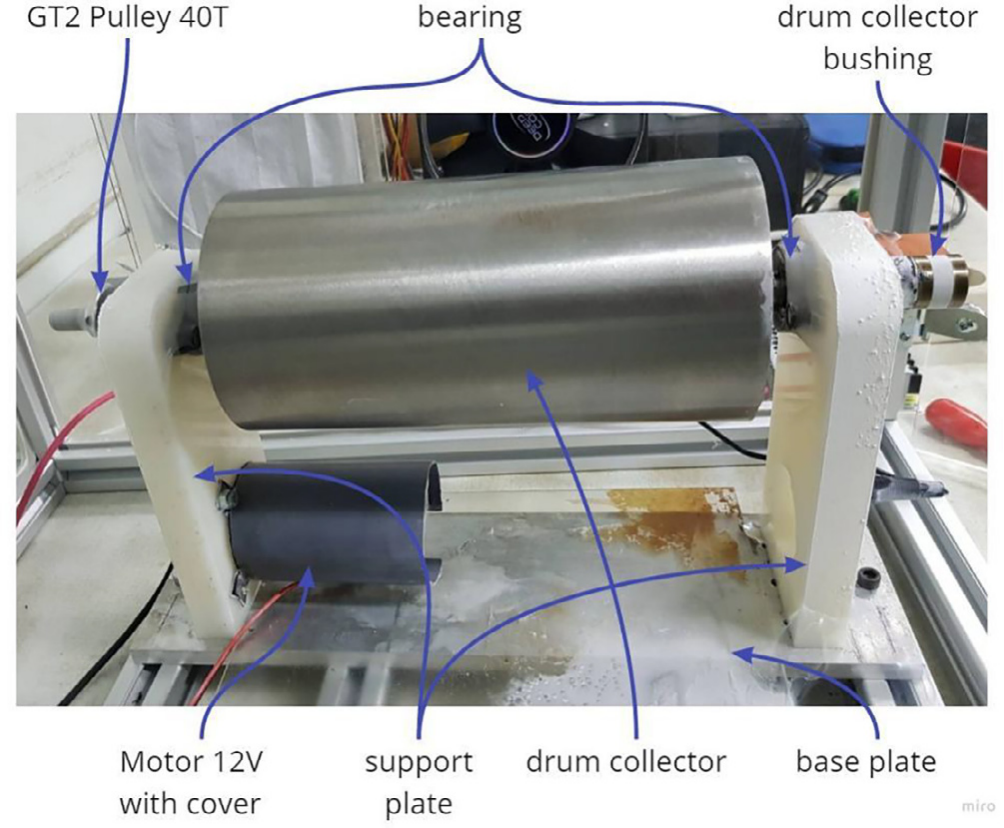
Drum collector components.

Fig. 4, Fig. 5 show the mechanical components of the syringe pump and drum collector. The manufacturing process employed is the cutting and drilling procedures with the aluminum profile purchased and cut to the desired size. The support, motor mount and base plates are made of aluminum or nylon materials, with the machining process carried out in a laboratory. The area adjacent to the high voltage source consisting of the syringe holder and drum collector support plate is made of nylon materials to prevent the flow of electric current to the base frame. The designs or drawings of the plates used in this machine are shown in Table 2.

**Table 2:** Design files.

Design file name	File type	Open-source license	Location of the file
*Syringe pump “aluminum profile 30 × 30 × 150”*	*Autodesk Inventor CAD file*	*CC BY 4.0*	DOI: 10.17632/vzmd24hzhm.1
*Syringe pump “Support plate.”*	*Autodesk Inventor CAD file*	*CC BY 4.0*	DOI: 10.17632/vzmd24hzhm.1
*Syringe pump “top pusher plate.”*	*Autodesk Inventor CAD file*	*CC BY 4.0*	DOI: 10.17632/vzmd24hzhm.1
*Syringe pump “bottom pusher plate.”*	*Autodesk Inventor CAD file*	*CC BY 4.0*	DOI: 10.17632/vzmd24hzhm.1
*Syringe pump “Bottom Syringe Holder.”*	*Autodesk Inventor CAD file*	*CC BY 4.0*	DOI: 10.17632/vzmd24hzhm.1
*Syringe pump “Top Syringe Holder.”*	*Autodesk Inventor CAD file*	*CC BY 4.0*	DOI: 10.17632/vzmd24hzhm.1
*Syringe pump “Side Motor Holder.”*	*Autodesk Inventor CAD file*	*CC BY 4.0*	DOI: 10.17632/vzmd24hzhm.1
*Syringe pump “Motor Holder.”*	*Autodesk Inventor CAD file*	*CC BY 4.0*	DOI: 10.17632/vzmd24hzhm.1
*Syringe Pump Assembly*	*Autodesk Inventor CAD file*	*CC BY 4.0*	DOI: 10.17632/vzmd24hzhm.1
*Drum Collector*	*Autodesk Inventor CAD file*	*CC BY 4.0*	DOI: 10.17632/vzmd24hzhm.1
*Drum Collector Base Plate*	*Autodesk Inventor CAD file*	*CC BY 4.0*	DOI: 10.17632/vzmd24hzhm.1
*Drum Collector Support Plate*	*Autodesk Inventor CAD file*	*CC BY 4.0*	DOI: 10.17632/vzmd24hzhm.1
*Drum Collector Bushing*	*Autodesk Inventor CAD file*	*CC BY 4.0*	DOI: 10.17632/vzmd24hzhm.1
*Drum Collector Assembly*	*Autodesk Inventor CAD file*	*CC BY 4.0*	DOI: 10.17632/vzmd24hzhm.1
*Base Frame Assembly*	*Autodesk Inventor CAD file*	*CC BY 4.0*	DOI: 10.17632/vzmd24hzhm.1
*Box Cover Assembly*	*Autodesk Inventor CAD file*	*CC BY 4.0*	DOI: 10.17632/vzmd24hzhm.1
*Drum Collector electronic schematic*	*Schematic Fritzing*	*CC BY 4.0*	DOI: 10.17632/vzmd24hzhm.1
*Syringe Pump electronic schematic*	*Schematic Fritzing*	*CC BY 4.0*	DOI: 10.17632/vzmd24hzhm.1
*Rotary Drum Arduino Code*	*Arduino Code*	*CC BY 4.0*	DOI: 10.17632/vzmd24hzhm.1
*Syringe Pump Arduino Code*	*Arduino Code*	*CC BY 4.0*	DOI: 10.17632/vzmd24hzhm.1

### Electrical components

**Fig. 6:**
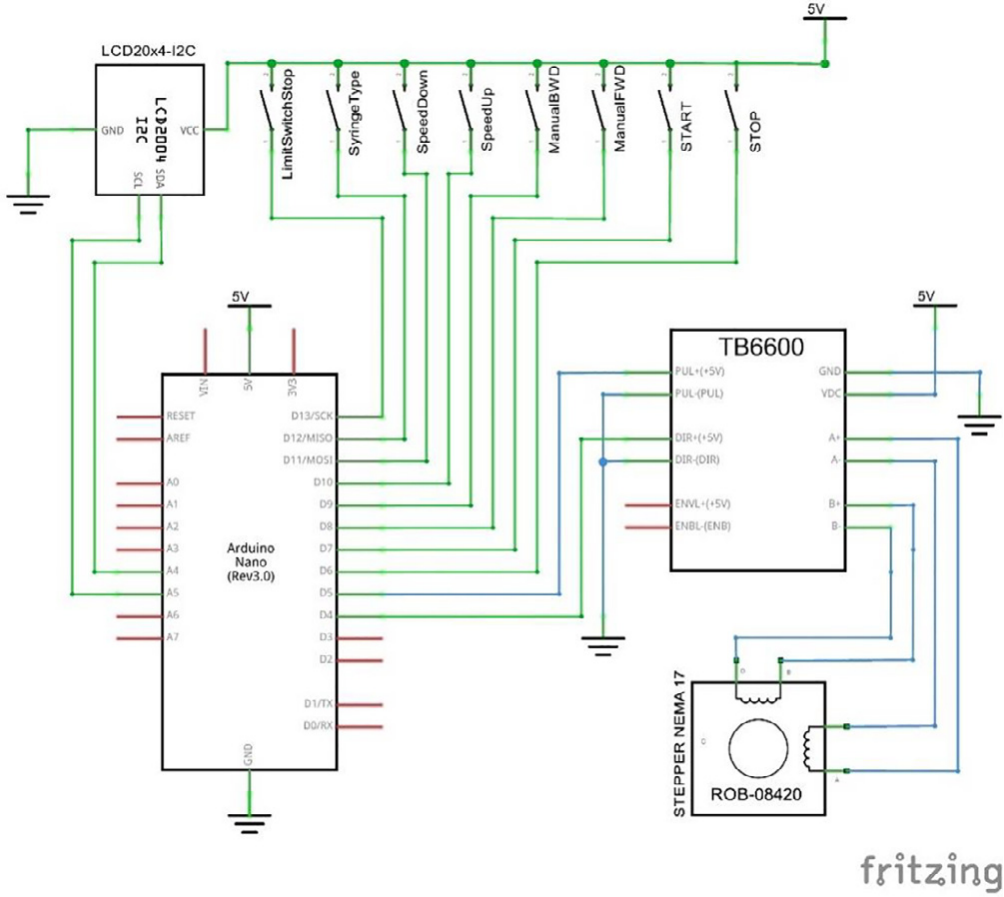
Electrical schematic of syringe pump controller.

**Fig. 7:**
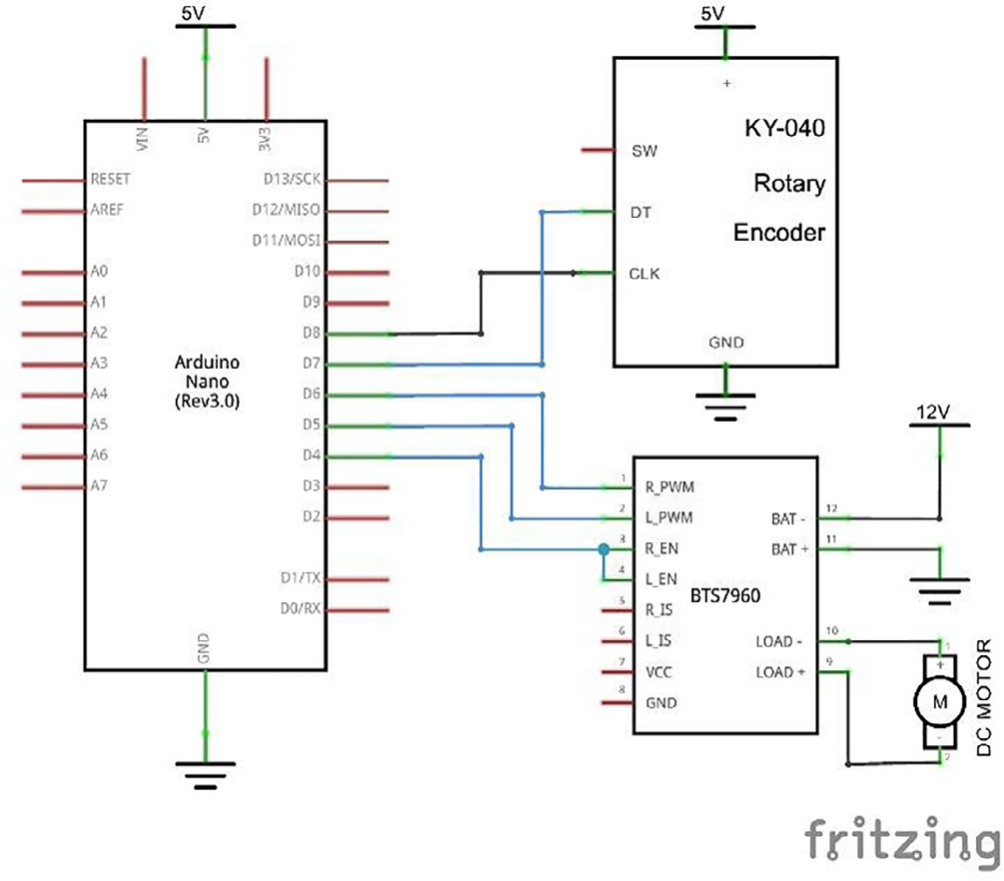
Electrical schematic of drum collector controller.

The electrical components of the syringe pump and drum collector are shown in Fig. 2, Fig. 3, while the schematic design of the electrical circuit is presented in Fig. 6, Fig. 7. The schematic design was made simple, hence, it easily connects the Arduino Nano with the buttons mounted on the control panel and actuators and sensors on the mechanical system. The control panel is used to adjust the feeding and rotational speed of the syringe pump and Drum Collector, respectively.

### Arduino nano software

**Fig. 8:**
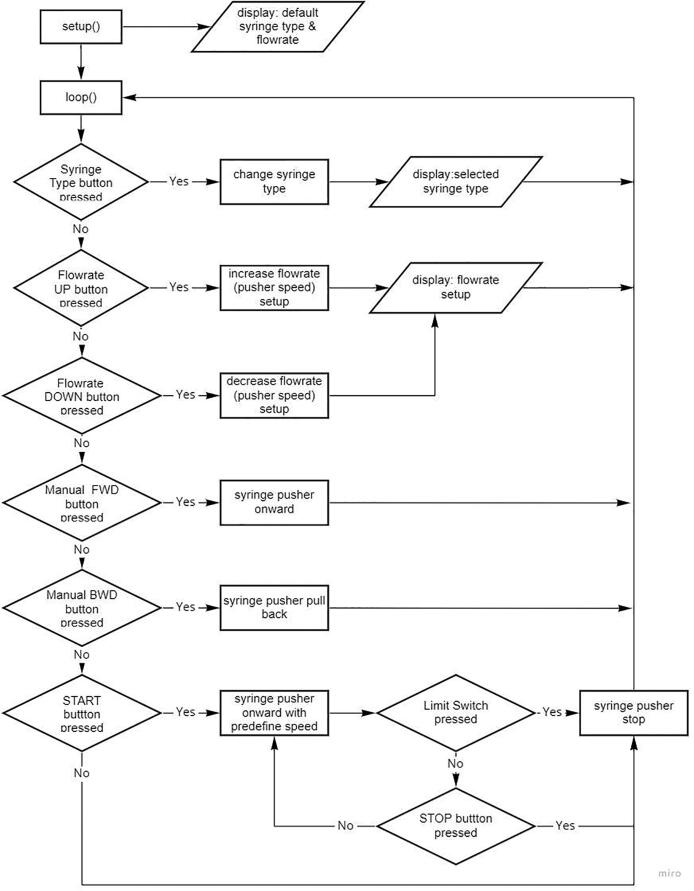
A simplified flow-chart showing the main functions of the syringe pump controller.

**Fig. 9:**
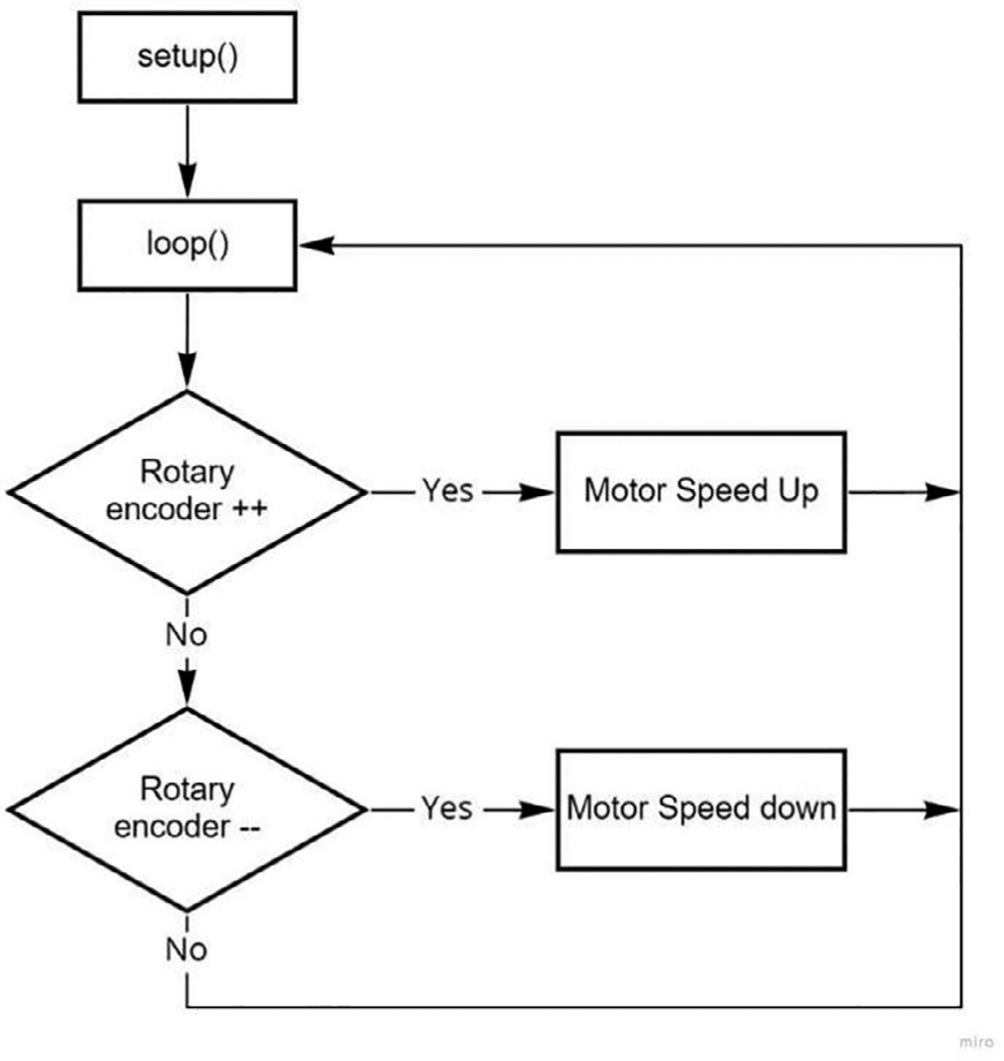
A simplified flow-chart showing speed controller of drum collector.

The software algorithm flow relating to the control and adjustment functions of the syringe pump and drum collector is shown in Fig. 8, Fig. 9. In the syringe pump setting function, the users are permitted to select the syringe size and the desired flow speed. Afterwards, they tend to advance and reverse the plunger until it touches the tip of the plunger, then the START button is pressed to start the process, which stops the syringe pump. The rotary encoder panel is rotated clockwise to increase its speed and vice versa.

### Design files summary

The design files of the in-house machine electrospinning can be seen in Table 2.

This machine is also equipped with a lid that can be opened and closed with a hinged door made of lightweight acrylic, very easy and practical when the machine is moved or cleaned after use. Indeed, the machine has a minimum risk of work accidents, because the risk of the operator to be electrocuted is smaller.

## Bill of materials

The bill of materials can be seen in Table 3.

### Bill of materials

**Table 3:** Bill of Materials.

Designator	Component	Number	Cost per unit USD	Total cost - USD	Source of Materials	Material Type
**Syringe Pump**						
Syringe pump “aluminum profile 30 × 30 × 150”	Aluminum Profile 30 × 30 × 150	4	1.05	4.20	Tokopedia	Aluminum
Syringe pump “Support plate.”	Support plate 150 × 160 × 15	2	56.00	112.00	Local retailer	Aluminum
Syringe pump “top pusher plate.”	Top pusher plate	1	10.50	10.50	Local retailer	Aluminum
Syringe pump “bottom pusher plate.”	Bottom pusher plate	1	10.50	10.50	Local retailer	Aluminum
Syringe pump “Bottom Syringe Holder.”	Bottom Syringe Holder	1	4.90	4.90	Local retailer	Nylon/polymer
Syringe pump “Top Syringe Holder.”	Top Syringe Holder	1	3.50	3.50	Local retailer	Nylon/polymer
Syringe pump “Side Motor Holder.”	Side Motor Holder plate	2	7.00	14.00	Local retailer	Aluminum
Syringe pump “Motor Holder.”	Motor Holder plate	1	7.00	7.00	Local retailer	Aluminum
Stepper Motor-Syringe pump	Nema 17HS4401 with PG 518	1	32.20	32.20	Tokopedia	Composite
Lead Screw-Syringe pump	Lead Screw (Slider Bar) D8x1mm	1	4.90	4.90	Tokopedia	Steel
Bearing-Syringe pump	KFL08 Bracket Bearing Shaft 8 mm	2	1.61	3.22	Tokopedia	Steel
Linear Bearing-Syringe pump	Linear Bearing SCS 10 UU (diameter 10 mm)	2	2.35	4.69	Tokopedia	Steel
Slider Bar Shaft-Syringe pump	Slider Bar Shaft D10mm	2	2.45	4.90	Tokopedia	Steel
NUT Seat-Syringe pump	NUT Seat T8	1	4.34	4.34	Tokopedia	Aluminum
Knob-Syringe pump	Knob M6	2	1.26	2.52	Tokopedia	Polymer
M3x15 screw	M3x15 screw bolts	8	0.07	0.56	Tokopedia	Steel
M4x20 screw	M4x20 screw bolts	32	0.11	3.36	Tokopedia	Steel
M4x12 screw	M4x12 screw bolts	20	0.08	1.68	Tokopedia	Steel
M6x25 screw	M6x25 screw bolts	6	0.21	1.26	Tokopedia	Steel
M6x50 screw	M6x50 screw bolts	2	0.35	0.70	Tokopedia	Steel
GT2 Pulley 30T	GT2 Pulley 30T, 8 mm bore	1	2.45	2.45	Tokopedia	Aluminum
GT2 Pulley 20T	GT2 Pulley 20T, 5 mm bore	1	0.88	0.88	Tokopedia	Aluminum
Timing Belt GT2	Timing Belt GT2 180 mm	1	0.84	0.84	Tokopedia	Rubber

**Sub Total**				**235.10**		

**Drum Collector**						
Drum Collector Base Plate	Drum Collector Base Plate	1	35.00	35.00	Local retailer	Aluminum
Drum Collector Support Plate	Drum Collector Support Plate	2	14.00	28.00	Local retailer	Nylon/ polymer
Drum Collector	Drum Collector	1	105.00	105.00	Local retailer	stainless steel
Drum Collector Bushing	Drum Collector Bushing Brass	1	7.00	7.00	Local retailer	Brass
Bearing KFL000	Bearing KFL000 (Bore 10 mm)	2	2.24	4.48	Tokopedia	Steel
Motor DC 12v	Motor DC 12v Type RS775	1	10.50	10.50	Tokopedia	Composite
GT2 Pulley 40 T	GT2 Pulley 40 T, 10 mm bore	1	3.15	3.15	Tokopedia	Aluminum
GT2 Pulley 20 T	GT2 Pulley 20 T, 5 mm bore	1	0.88	0.88	Tokopedia	Aluminum
Timing Belt GT2	Timing Belt GT2 220 mm	1	0.84	0.84	Tokopedia	Rubber
Acrylic Ruler 2 mm	Acrylic Ruler 2 mm	1	2.80	2.80	Tokopedia	Acrylic
M6x25 screw	M6x25 screw bolts	6	0.21	1.26	Tokopedia	Steel
M4x12 screw	M4x12 screw bolts	4	0.08	0.34	Tokopedia	Steel

**Sub Total**				**199.24**		

**Base Frame**						
Aluminum Profile 30 × 30 × 580	Aluminum Profile 30 × 30 × 580	4	3.64	14.56	Tokopedia	Aluminum
Aluminum Profile 30 × 30 × 520	Aluminum Profile 30 × 30 × 520	5	4.06	20.30	Tokopedia	Aluminum
Aluminum Profile 30 × 30 × 300	Aluminum Profile 30 × 30 × 300	2	2.10	4.20	Tokopedia	Aluminum
Angle Bracket	Angle Bracket	4	0.41	1.62	Tokopedia	Aluminum
Inner Bracket	Inner Bracket	4	1.26	5.04	Tokopedia	Aluminum
M6x12 screw bolts	M6x12 screw bolts	16	0.07	1.12	Tokopedia	Steel
End Cap	End Cap	4	0.46	1.82	Tokopedia	Polymer
Sliding Nut	Sliding Nut	4	0.24	0.95	Tokopedia	Steel

**Sub Total**				**49.62**		

**Box Cover**						
Aluminum Profile 30 × 30 × 750	Aluminum Profile 30 × 30 × 750	4	5.25	21.00	Tokopedia	Metal
Aluminum Profile 30 × 30 × 500	Aluminum Profile 30 × 30 × 500	8	3.50	28.00	Tokopedia	Metal
Solid right-angle connector	Solid right-angle connector	8	2.10	16.80	Tokopedia	Metal
Acrylic 760 mm × 510 mm	Acrylic 760 mm X510 mm	2	12.25	24.50	Tokopedia	Polymer
Acrylic 380 mm × 510 mm	Acrylic 760 mm X510 mm	2	6.65	13.30	Tokopedia	Polymer
Acrylic 510 mm × 510 mm	Acrylic 510 mm X510 mm	2	8.40	16.80	Tokopedia	Polymer
Magnetic Lock	Magnetic Lock	2	0.70	1.40	Tokopedia	Other
Hinge	Hinge	4	0.70	2.80	Tokopedia	Metal
Rubber seal	Rubber seal	1	3.50	3.50	Tokopedia	Rubber
Hand Handle	Hand Handle	2	1.00	2.00	Tokopedia	Metal
Fan	DEEPCOOL XFAN 120	2	4.20	8.40	Tokopedia	Metal
M6x12 screw bolts	M6x12 screw bolts	20	0.07	1.40	Tokopedia	Steel

**Sub Total**				**124.60**		

**Power Supply, Instrumentation and Control**						
Rotary Encoder	Rotary Encoder	1	16.80	16.80	Tokopedia	Other
Arduino Nano	Arduino Nano	2	7.00	14.00	Tokopedia	Other
Pulse Meter Encoder	Pulse Meter Encoder	1	21.00	21.00	Tokopedia	Other
Proximity sensor	PR08 2DP 2 mm PNP 12–24 V Proximity Sensor	1	8.75	8.75	Tokopedia	Other
LCD 20 × 4	LCD 20 × 4 Blue Backlight with I2C	1	4.20	4.20	Tokopedia	Other
LCD 16 × 2	LCD 16x2 Blue Backlight with I2C	1	2.10	2.10	Tokopedia	Other
Motor Driver BTS7960	Motor Driver BTS7960 H-bridge	1	8.05	8.05	Tokopedia	Other
TB6600S Motor Driver	TB6600 4.5A Stepper Motor Driver	1	10.15	10.15	Tokopedia	Other
Power Supply 12 V, 4A	Meanwell Power Supply LRS-50–12	1	12.60	12.60	Tokopedia	Other
Power Supply 12 V, 8A	Meanwell Power Supply LRS-100–12	1	19.67	19.67	Tokopedia	Other
Cable and connector	Cable and connector	10	1.40	14.00	Tokopedia	Other
PCB	PCB	2	3.50	7.00	Tokopedia	Other
Box Electronic Instrument Project	Box Electronic Instrument Project	1	17.50	17.50	Tokopedia	Plastic
High Voltage Power Supply	High Voltage Power Supply up to 30 KV	1	847.00	847.00	AliExpress	Other

**Sub Total**				**1,002.82**		

**Total Cost**				**1,611.37**		

## Build instructions

### Syringe pump build instruction

The first process involved in manufacturing a syringe pump is the purchase of components, listed in the bill of material, as shown in Table 3. The 30 × 30 aluminum profile is cut into 4 pieces with a length of 150 mm and then inserted in 4 small holes at the ends, with Screw Tapper M4. The support, upper and lower pusher plates, lower and upper syringe holders, side motor mount and motor mount components are produced by machining according to the designs on the online repository. Furthermore, when all the parts have been completed, then the assembly process is carried out by installing all the components and their bolts into a system, as shown in Fig. 4. The next step is to install the cables and connectors to connect it to the control panel.

### Drum collectors build instruction

The initial stage is to carry out the machining process to produce the base and support plates, drum, and bushing components. Interestingly, of the several manufacturers, this is the most difficult where the eccentricity of the shaft and drum cylinder, including its balance, needs to be considered. This is necessary because it enables the drum to rotate properly without significant vibration. The next step is to assemble all components in Table 3 to obtain the system shown in Fig. 5. Installation of the rotary encoder, 12-volt DC motor power cable and High Voltage supply is required at the final stage.

### Base frame and cover box build instruction

**Fig. 10:**
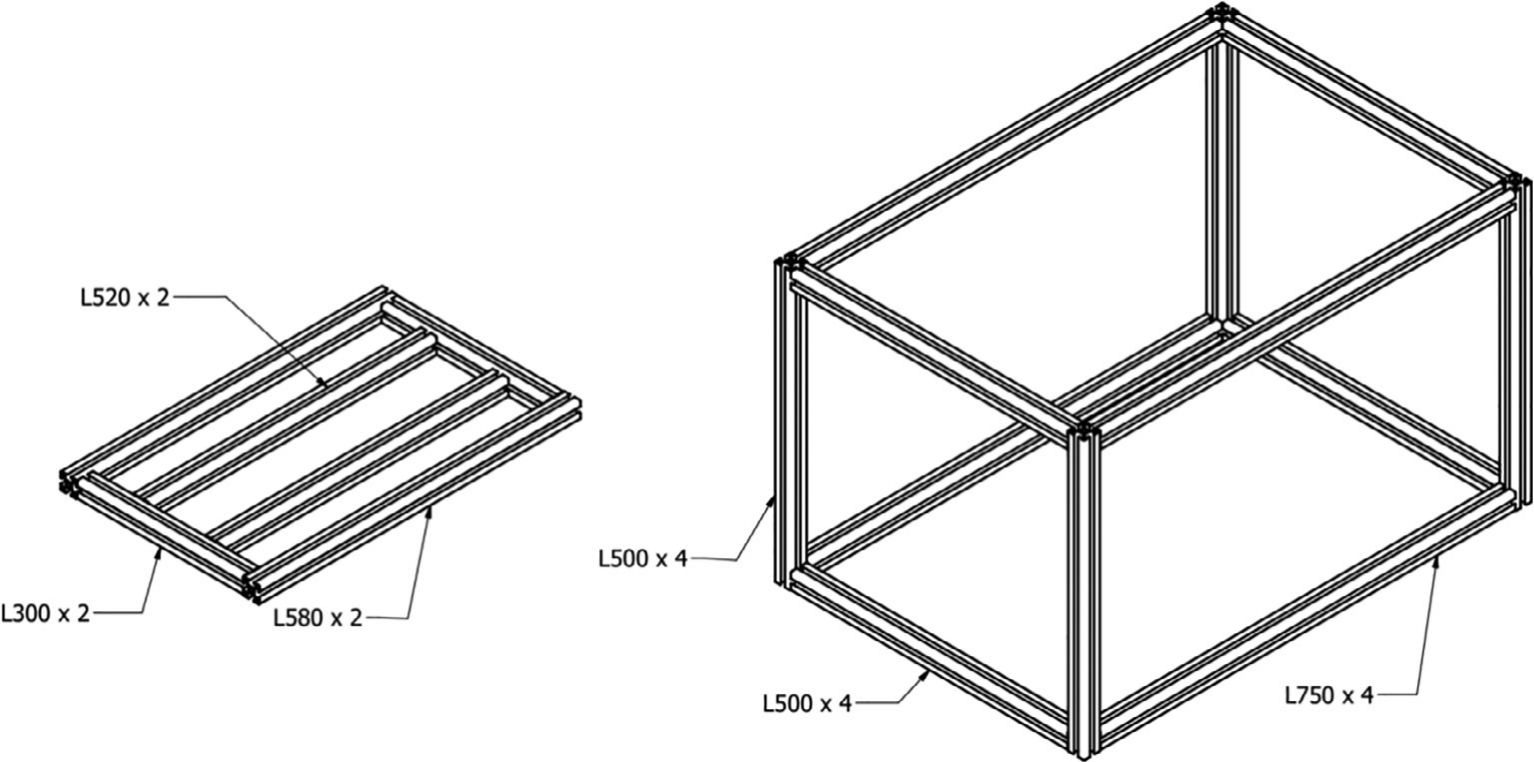
Assembly of the base frame (left) and the box cover (right).

When the Syringe Pump and drum collector have been completed, the next step is to assemble the base frame consisting of the 30 × 30 aluminum profile pieces listed on the bill of materials in Table 3. These are connected to the angel and inner brackets, locked with bolts to form a base frame, as shown in Fig. 10. In circumstances where these have been made rigid, the subsequent step is to install the syringe pump and drum collector on the base frame, which is locked with bolt connections to enable it to stand firmly.

It is necessary to make a box cover with a frame to isolate and secure the electrospinning machine, as shown in Fig. 10. An acrylic sheet of the appropriate size for the bill cover box is further attached, thereby isolating the machine from the external environment, as shown in Fig. 1.

### Electrical or panel control build instruction

**Fig. 11:**
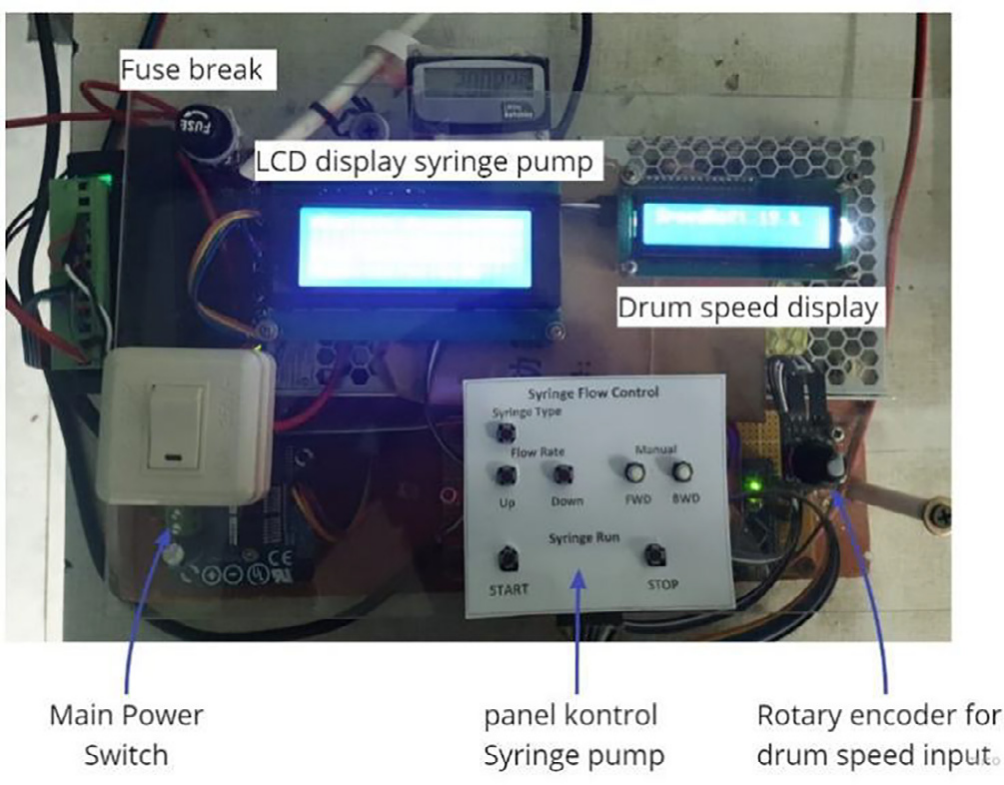
Front view of the control panel.

To fabricate an electrical control system, the first step is to adjust the position of the Arduino nano, power supply and motor driver on the box panel, then connect them using connectors and cables according to the schematic images in Fig. 6, Fig. 7. The power supply to the syringe pump and drum collector are separated, therefore, when a short circuit occurs in one system, it does not damage the entire connection. A fuse is installed in this electrical system to prevent fires in electronic components as additional safety. The front view of the control panel box is shown in Fig. 11.

## Operation instructions

1. Prepare a solution using a nanofiber material and then put it in a syringe.
2. Ensure all power supplies are off, and then attach the syringe containing the solution to its holder by loosening the lock knob and then tightening it again. Afterwards, attach the positive connector of the high-voltage supply to the syringe.
3. Attach the aluminum foil to the drum collector using double-sided tape and affix the negative high-voltage supply connector to its pole.
4. Turn on the main control panel switch to select the type of syringe size used and inject the solution into the nanofiber fabrication process while adjusting the plunger position to stick to the end.
5. Adjust the rotation of the drum collector to the desired speed check that the aluminum foil does not come off the drum.
6. Assuming the drum is properly rotated, and no aluminum foil was released, turn on the high voltage supply and adjust as desired. The nanofiber fabrication begins by pressing the Start button on the syringe pump control panel.
7. Immediately the solution in the syringe is used up, press the stop button on the control panel, and turn off the rotating drum using the dial. Then, turn off the high-voltage supply and remove the positive charge connector previously clamped on the syringe.
8. Carefully remove the previously attached aluminum foil to collect the produced nanofiber.

## Validation and characterization

In order to ensure that a high-quality nanofiber can be produced, it is necessary to validate machine performance by performing an evaluation on the properties of the resulting nanofiber. A series of techniques including Scanning Electron Microscopy (SEM) for microstructural analysis using HITACHI (HITACHI FLEXSEM 100), X-ray powder diffraction (XRD) for phase structural interpretation using X-Pert Pro-MPD, Fourier Transform Infrared Spectroscopy (FTIR) for chemical bonds quantification using Nicolet iS 10 FTIR Spectrometer, Thermo Gravimetric Analysis (TGA) and Differential Scanning Calorimetry (DSC) for material’s thermal stability and weight loss analysis during the heating at constant rate 10 °C/min using LINSEIS STA PT 1600, tensile tests on Computer Servo Control Material Testing Machine for measuring mechanical properties using Hung Ta Instrument – 8160 were carried out to evaluate and validate the nanofiber’s properties and quality. Some similarities were detected between the prepared and reviewed nanofibers. Moreover, the in-house electrospinning machine is proved to be more reliable and trustworthy. Otherwise, its re-manufacturing and re-development is an option.

When it comes to the morphology of the resulted nanofiber, the size of nanofiber can mainly be affected by three different factors, precursors, processes, and ambient factors according to [23]. When the dielectric properties and surface charge density of the precursors increase, the diameter of the nanofibers decreases. In terms of process factors, as the voltage increases, the nanofiber diameter decreases. However, decreasing the flow rate results in a decrease in the diameter of the nanofibers. In this case, in order to obtain thinner nanofiber, reducing polymer concentration and injection rate, increasing the collector speed, and relative humidity are preferred, while increasing the applied voltage helps the bead type formation.

### SEM images

**Fig. 12:**
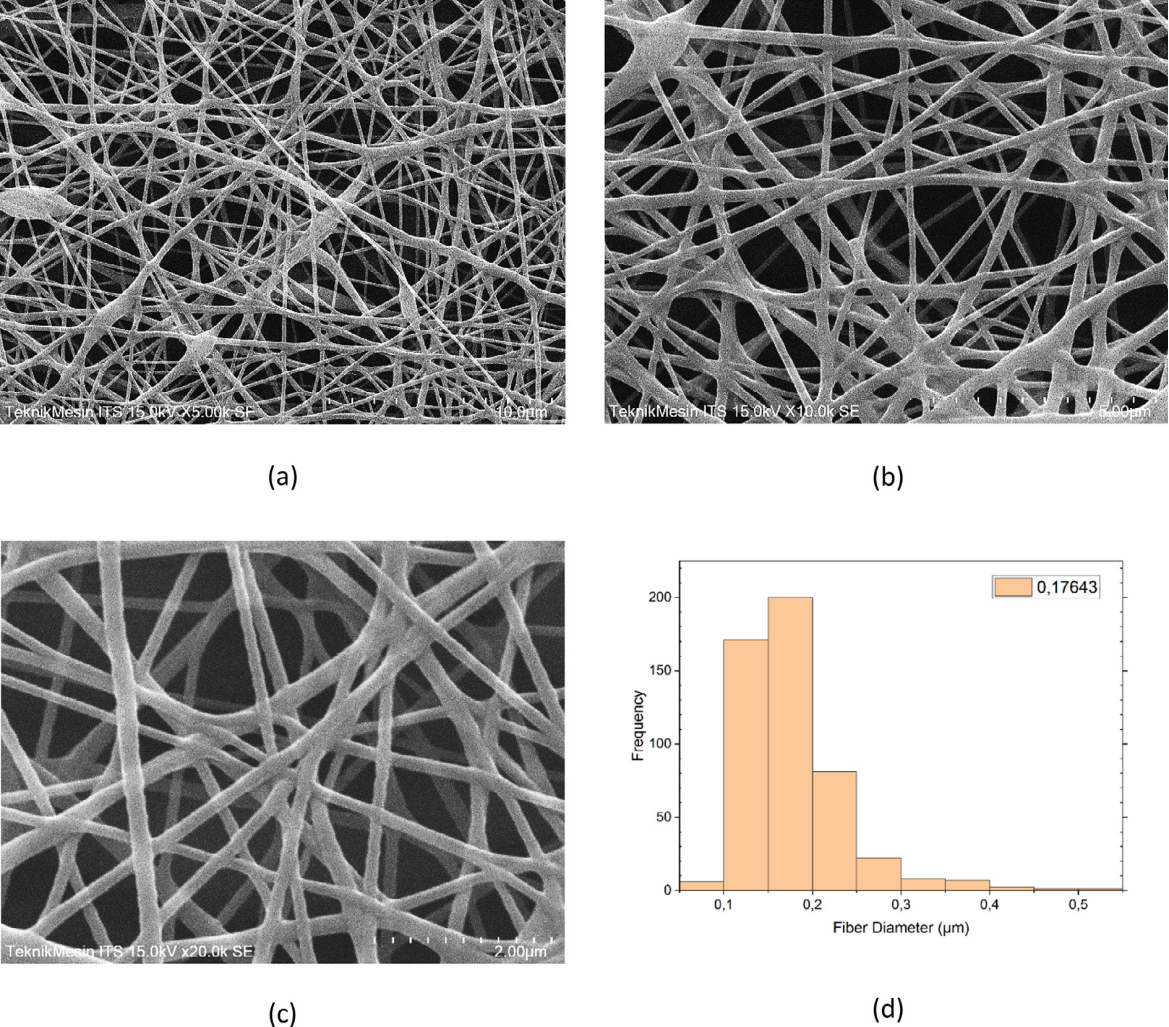
SEM images of PVA-based electrospun nanofiber with 5,000x (a), 10,000x (b), 20,000x (c) magnifications, and histogram for frequency of the diameter nanofiber (d). From those images, it is shown that the average diameter of nanofiber is approximately 170 nm.

The resulting SEM images of the electrospun nanofiber’s structure are shown in Fig. 12. It is worth noting that it was made thick enough thereby, making it easier to peel from the surface of the aluminum and afterwards inserted into the SEM chamber. Magnification of 5,000x, 10,000x and 20,000x were selected as shown in Fig. 12(a), (b), and (c) respectively to examine the size. Based on those images, it is evident that the diameter of the produced fiber is within the range of 10 nm and 500 nm. Several preliminary studies have also reported its size range [24], [25], [26], [27]. Fig. 12(d) shows the histogram of the average diameter of the nanofibers produced, which is 170 nm. In this case, the diameter of the nanofiber met the criteria to be classified as nanofiber.

**Fig. 13:**
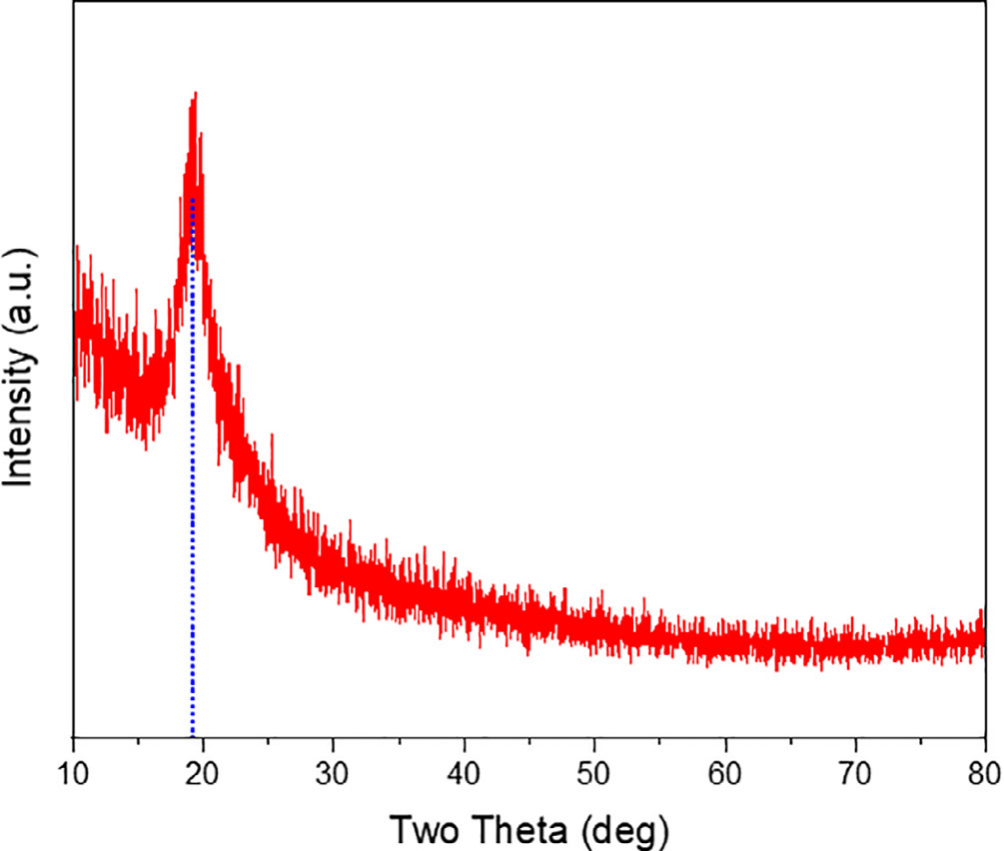
XRD patterns resulted from the characterization of the PVA based electrospun nanofiber with the peak at 19.3° of 2-theta.

The interconnecting pore is surrounded by the nanofiber shown in Fig. 13(a) with a varying size ranging within 19 nm and 183 nm. Beaded ones are also observed in Fig. 4(a) and (b), and its structure is strongly affected by various parameters used during the electrospinning process, namely, the applied voltage, polymer concentration and injection rate, tip-to-collector distance, rotation speed of the collector, and relative humidity. Reducing the polymer concentration and injection rate, increasing the rotation speed of the collector, and relative humidity is preferable to obtain thinner fibers, whereas increasing the applied voltage assist in the formation of the beaded types [28], [29], [30], [31], [32].

Fig. 12(d) shows that the resulting nanofiber has an average diameter of 170 nm with the largest frequency. The second largest frequency is 170 with a resulting diameter of 100–150 nm. For the largest diameter of 400–450 nm, the resulted frequency is less than 20. In general, with 500 times diameter calculation using ImageJ, the consistency of nanofiber morphology is quite good considering the mean diameter of nanofiber. The range of diameter nanofiber is sufficient. However, the nanofiber alignment is required to improve.

### XRD results

The XRD pattern of the electrospun nanofiber in Fig. 13 shows a semi-crystalline peak at 19.1° with FWHM of 0.2007, which is equal to 41.94 nm of crystallite size, much lower than reference [33]. In this case, the occurrence of strong inter- and intramolecular hydrogen bonds is one of the reasons [26], [27], [34]. Another reason is high molecular weight of PVA nanofiber which has superior crystalline properties [26], [27]. The XRD pattern of the electrospun nanofibers is in line with previous studies that stated in-house machine is successfully used to fabricate nanofibers with standard structure.

### FTIR results

**Fig. 14:**
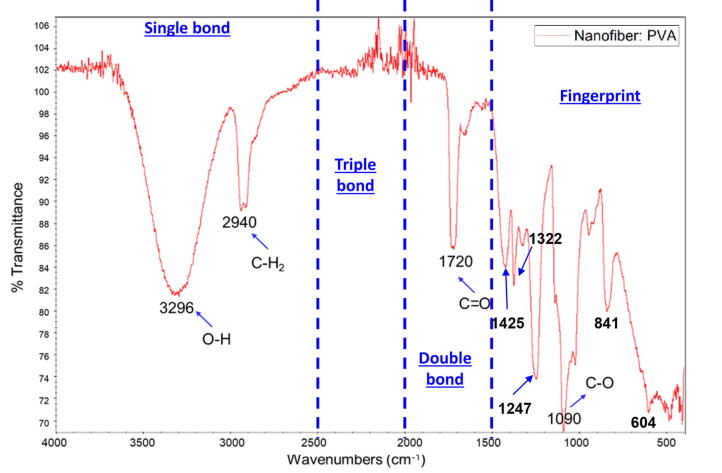
FTIR pattern of the studied material.

PVA material was detected from the functional groups pattern of resulted FTIR spectra, especially when observing the fingerprint groups which are only owned by PVA material. The FTIR pattern of the electrospun PVA nanofiber is shown in Fig. 14 containing four functional groups which include single bond area (4000–2500 cm^−1^ wavenumbers), triple bond area (2500–2000 cm^−1^ wavenumbers), double bond (2000–1500 cm^−1^ wavenumbers), and fingerprint area (<1500 cm^−1^ wavenumbers). The absorption peaks were clearly shown at 3296 cm^−1^ as O–H bonding resonance, 2940 cm^−1^ as CH_2_ bonding resonance, 1720 cm^−1^ as C═O bonding resonance, 1425 cm^−1^, 1322 cm^−1^, 1247 cm^−1^, 1090 cm^−1^, 841 cm^−1^, and 604 cm^−1^ as fingerprints resonances [13], [14], [15]. In this case, the product contains chemical PVA bonding free from impurities. This implies that the machine produces pure PVA polymer nanofiber.

### TGA/DSC results

**Fig. 15:**
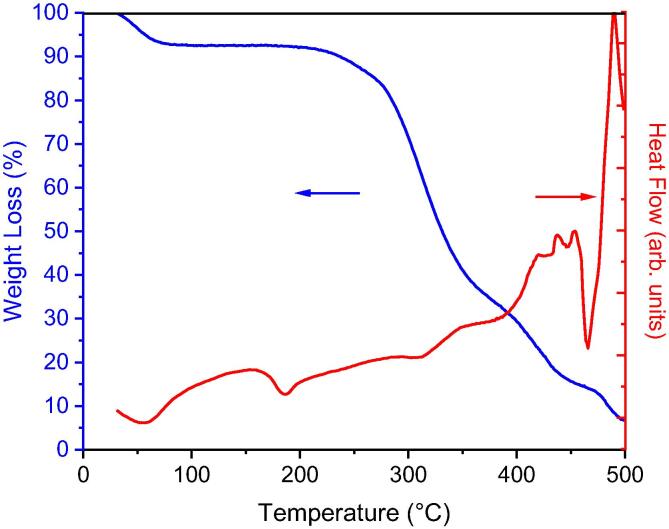
TGA (blue) vs. DSC (red) graphs of PVA electrospun nanofiber. (For interpretation of the references to colour in this figure legend, the reader is referred to the web version of this article.)

Thermal analysis characterization of TGA/DSC on PVA nanofibers was carried out at a constant rate of 10 °C/min using LINSEIS STA PT 1600. The TGA/DSC thermograms can be seen in Fig. 15 marked by blue and red solid lines respectively. The TGA curve contains three stages of decomposition of the electrospun PVA nanofiber. The first weight loss started at 43.2–63.2 °C with an endothermic peak of 72.2 °C corresponding to the removal of moisture from the absorbed water in the sample. The second weight lost occurred at 220.2–353.2 °C with an exothermic peak of 275.2 °C indicating the degradation of a polymer structure. Indeed, in the final stage above 360 °C with an exothermic peak at 478.2 °C, the polymer underwent massive decomposition to carbon oxides and volatile hydrocarbons. These results are in accordance with the previous research conducted by [35], [36][37].

### Tensile properties

**Fig. 16:**
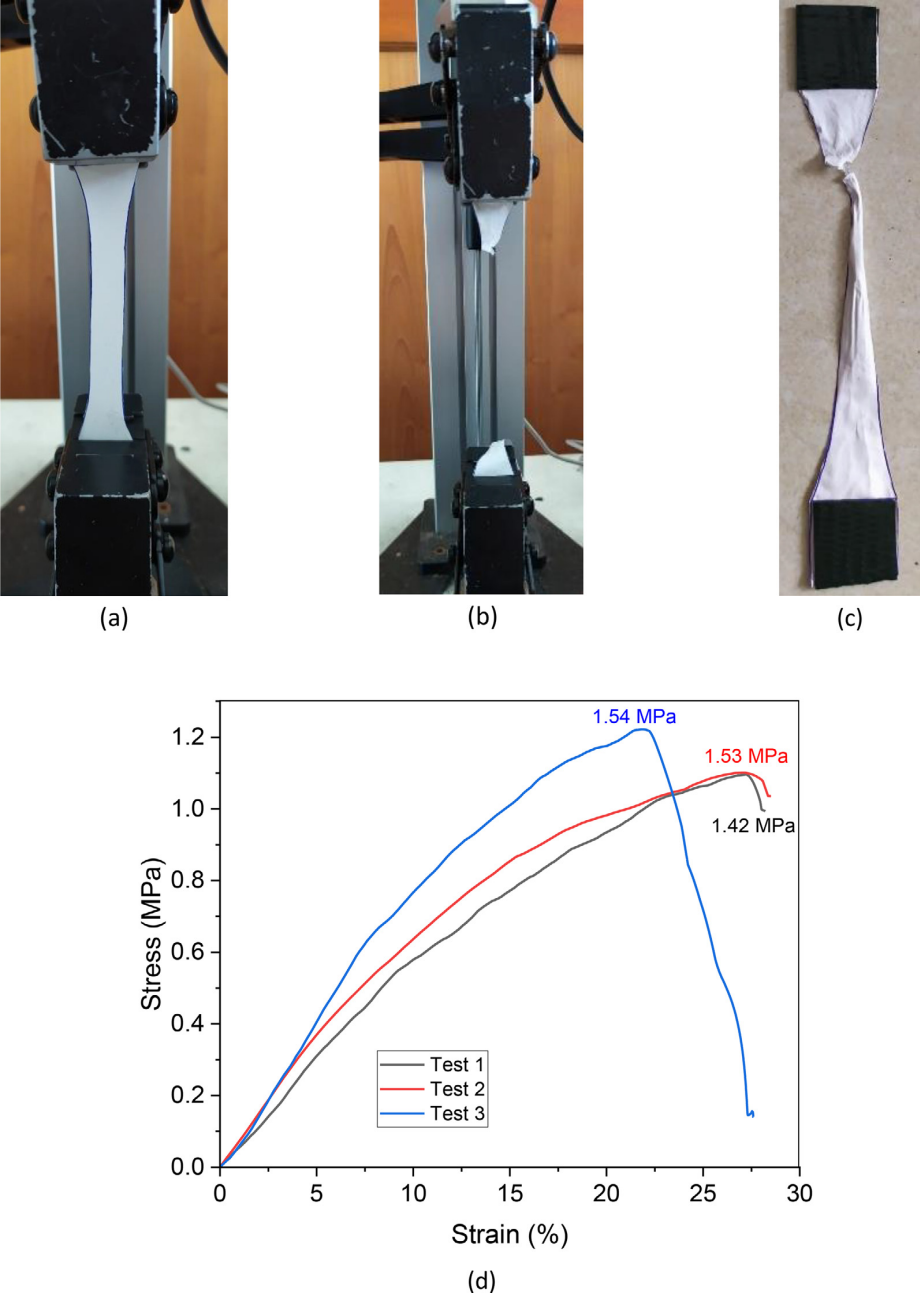
Tensile test on nanofiber specimens according to ASTM D638-14 at a rate of 2 mm/min, at the start of the test (a), when fracture (b), when released from the machine (c), and stress vs. strain curves for PVA nanofiber at constant speed of 2 mm/min (d).

According to ASTM D638-14, the resulting nanofiber web with a thickness of 0.2 mm was made into a standard specimen for uniaxial tensile measurements which can be seen in Fig. 16. Tensile tests were carried out three times to ensure reproducibility at a constant tensile test speed of 2 mm/min. The average value is taken from the three measurement results. Both ends are wrapped with tape to avoid slipping when the load is applied. Since the nanofiber web was collected using a drum collector containing a high conductivity polymer, it was rather difficult to obtain a perfect nanofiber web. In this case, the uniformity and consistency of the nanofiber web led to different tensile properties resulting from the experiments. Similarly, in this research, as seen in Table 4, the tensile strength, young modulus, and elongation of PVA nanofiber was only 1.49 MPa, 4.33 MPa, and 35.33% respectively. These results are quite different from others similar research [35], [38]. However, the type of polymer and its molecular weight also need to be considered in obtaining tensile properties.

**Table 4:** Tensile properties of electrospun PVA nanofiber.

Sample	Tensile strength (MPa)	Young modulus (MPa)	Elongation (%)
PVA nanofiber	1.49 ± 0.06	4.33 ± 0.47	35.33 ± 4.61

### Surface area

**Fig. 17:**
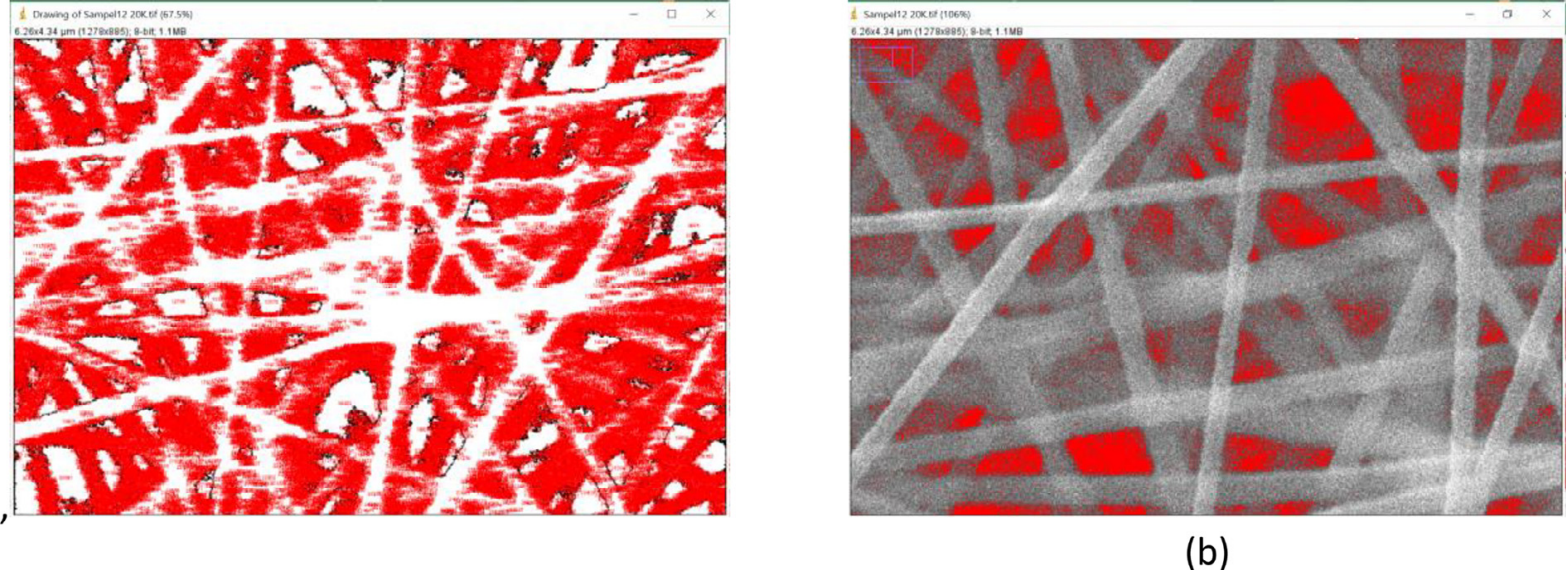
Measurement of surface area using ImageJ, background (a), nanofiber area (b).

The specific surface area can be approached using ImageJ analysis by measuring the background and subtracting the area from the total to get the total area of the nanofiber as seen In Fig. 17 [39], [40], [41]. The resulting surface area can be seen in Table 5. The ImageJ analysis is an approximation step to obtain the more accurate specific surface area results. However, ImageJ analysis are still welcome and acceptable in approaching the surface area results.

**Table 5:** Surface area of nanofiber.

Specimen No.	Total area [µm^2^]	Pores area [µm^2^]	Nanofiber area [µm^2^]
1	27.83	4.19	23.64
2	27.40	3.60	23.80
3	27.19	2.00	25.20

## Human and animal rights

None.

## CRediT authorship contribution statement

**Ika Dewi Wijayanti:** Conceptualization, Project administration, Writing – original draft. **Ari Kurniawan Saputra:** Software, Methodology, Validation, Supervision. **Faris Ibrahim:** Data curation, Investigation, Visualization. **Amaliya Rasyida:** Resources, Supervision, Validation. **Putu Suwarta:** Visualization, Writing – review & editing. **Indra Sidharta:** Methodology, Supervision, Writing – review & editing.

## Declaration of Competing Interest

The authors declare that they have no known competing financial interests or personal relationships that could have appeared to influence the work reported in this paper.
